# ‘I didn’t think you were allowed that, they didn’t mention that.’ A qualitative study exploring women’s perceptions of home birth

**DOI:** 10.1186/s12884-018-1733-1

**Published:** 2018-04-18

**Authors:** Jo Naylor Smith, Beck Taylor, Karen Shaw, Alistair Hewison, Sara Kenyon

**Affiliations:** 1 Care Quality Commission, Citygate, Gallowgate, Newcastle upon Tyne, NE1 4PA England, UK; 2 Institute of Applied Health Research, University of Birmingham, Birmingham, B15 2TT England, UK; 3 Institute of Clinical Sciences, University of Birmingham, Birmingham, B15 2TT England, UK

**Keywords:** Choice behaviour, Delivery, Obstetric, Home childbirth, Maternal behaviour, Midwifery, Patient satisfaction, Personal Autonomy, Pregnancy, Prenatal care

## Abstract

**Background:**

Evidence suggests that home birth is as safe as hospital birth for low risk multiparous women, and is associated with reduced intervention rates and increased rates of normal birth. However the home birth rate in the UK is low, and few women choose this option. The aims of this study were to identify what influences multiparous women’s choice of birth place, and to explore their views of home birth.

**Methods:**

Five focus groups were conducted with multiparous women (*n* = 28) attending mother and baby groups in a city in the UK with a diverse multi-ethnic population. Data were analysed thematically using the Framework Method, combining deductive and inductive approaches to the data.

**Results:**

Several themes were developed from the data, these were: the expectation that birth would take place in an Obstetric Unit; perceptions of birth as a ‘natural’ event; lack of knowledge of what home birth looked like; and a lack of confidence in the reliability of the maternity service. Two themes emerged regarding the influences on women’s choices: clear information provision, particularly for those from ethnic minority groups, and the role of health care professionals. A final theme concerned women’s responses to the offer of choice.

**Conclusions:**

There are gaps in women’s knowledge about the reality and practicalities of giving birth at home that have not been previously identified. Other findings are consistent with existing evidence, suggesting that many women still do not receive consistent, comprehensive information about home birth. The findings from this research can be used to develop approaches to meet women’s information and support needs, and facilitate genuine choice of place of birth.

**Electronic supplementary material:**

The online version of this article (10.1186/s12884-018-1733-1) contains supplementary material, which is available to authorized users.

## Background

Recent evidence suggests that home birth is as safe as hospital birth for low risk multiparous women, and that planned birth at home is associated with reduced intervention rates and increased rates of normal birth [1–5]. In addition, economic analyses indicate that home birth is a cost-effective option [6, 7]. As a result many care providers now promote home birth for low risk multiparous women. The recent National Maternity Review in England recommended that women are offered a genuine choice of birth place options [8], and the National Institute for Health and Care Excellence in the UK recommends that home birth is offered as an option to multiparous women at low risk of complications [1]. However, home birth is rare in England, at a rate of just 2.3% in 2015, [9], and rates are similarly low in other high income countries [10–13].

The birth rate in England has increased dramatically in recent years: between 2001 and 2012 the number of live births increased by 23% [14]. This, along with an increase in the incidence of complex pregnancies, has resulted in greater demand for birth in obstetric or Midwife-led maternity units. If more low risk multiparous women gave birth at home, it would increase hospital capacity for women at higher risk, and reduce intervention and caesarean section rates [15]. A recent evidence review synthesised the findings from 20 studies to determine what is known about women’s birth place preferences and decision-making in the UK [16]. It found the key influences on women’s decisions about place of birth included: receipt of information about the right to choose birth place, and the options available; personal preferences for different services; beliefs about safety and risk; prior birth experience; and information provided by family, friends and healthcare professionals. The study reported here presents findings concerning the views of women living in a large multi-ethnic city in England of home birth choice. The evidence synthesis was undertaken subsequent to the empirical work presented in this paper, and while many of the findings align, this study has elicited novel findings regarding women’s awareness of what home birth involves, and the extent to which health services meet their? information needs.

## Method

The aims of this qualitative study were to identify the factors that influence women’s choice of place of birth, and to explore their views of home birth. The study was designed to examine these issues specifically from the perspective of low risk multiparous women, as this group is likely to experience the most benefit from home birth.

A qualitative focus group study was conducted between May and June 2014 in a specific geographical area of a large multi-ethnic city in the UK served by a maternity service provider. Ethical approval was obtained from the NHS Research Ethics South Central Committee B (reference 14/SC/1007). We took a broadly interpretive approach which involved seeking culturally derived and historically situated interpretations of the social life-world [17]. Five focus groups were held during routine mother and baby (0–1 year) groups in Children’s Centres in the area of the city designated for the study. These groups were purposively selected to reflect the ethnic and social diversity of the area, and to include groups that were well-attended [18]. Some groups were ethnically diverse, while others were homogeneous. Women attending were by definition multiparous, and may have been considering birth place options for subsequent pregnancies.

JNS identified and recruited ‘gate keepers’ responsible for running the mother and baby groups who distributed study information a week before the focus groups were planned. While selection of the groups was purposive, a convenience sampling approach was taken for individual participants, with women attending the mother and baby groups invited to participate [18]. Data regarding the exact number of non-participants was not gathered, as access to participants was via a professional ‘gatekeeper’ and if women declined to take part they had no further contact with the researchers. Most or all of the women attending the mother and baby groups took part.

The focus groups lasted around one hour, were facilitated by JNS, and moderated by KS and BT. The focus group questions were developed by the research team using the findings from a supporting literature review exploring women’s perceptions. This is provided as a Additional file 1. Questions covered knowledge about birth place options, the role of professionals and family in birth place choices, priorities for birth place choice, views of the advantages and disadvantages of home birth, and information needs for home birth. The focus groups were digitally recorded and transcribed verbatim, and supplemented with contemporaneous field notes. Researchers explained their role and the research purpose prior to data collection and written consent was obtained.

### Analysis

Management and analysis of the data was conducted using the Framework Method [19], involving the following steps: anonymisation of data; familiarisation with the data; deductive analysis of two transcripts using codes identified following the review of the existing evidence; inductive, ‘open coding’ of these two transcripts to identify new, emergent themes; developing themes from coding labels; using these themes to develop an analytical framework; combining the deductive codes and inductive analytical framework and applying this to the remaining data; charting the data into a matrix; interpreting the charted data. A combined deductive and inductive approach to analysis was taken. Specific themes identified in the literature were identified deductively, whilst allowing for other elements of the women’s experience to emerge [20]. Table 1 presents the analytical framework.

**Table 1:** Analysis Framework

Analytic theme	Descriptive theme	Codes	Description
Lack of knowledge about what home birth looked like	What does home birth look like?	Familiarity Experience	- (Limited) Familiarity with concept of home birth - Perceptions that homebirth may influence experience (i.e. potential offers a better experience, but home linked to negative associations if things ‘go wrong’)
		Practicalities	- Uncertainty about the practicality, cost, consequences of home-birth
		Aftercare	- Concerns about reduced postnatal care after a home birth
Expectation of birth in an OU	Assumption’ that they would give birth in an OU	OU ‘normal’ Cultural norms	- OU described as ‘normal’/‘usual’ place of birth - Participants describe making decisions in reference to cultural norms
		Option/choice	- Homebirth is an option rarely (or not) offered/discussed
	OU perceived as safer than home	High Risk OU safe Managing emergencies	- Birth described as carrying significant risk - OU described as safer option compared to home birth (minimizes risk) - OU considered to have greater ability to respond to emergencies/provide specialist staff
Lack of confidence in the reliability of the maternity service	Confidence in service at time of birth	Safety	Concerns about ability of maternity services to provide - safe birth at home
		Responsiveness	Concerns about ability of maternity service to be - available for home birth when required
		Accessibility	- Concerns about ability of maternity services to provide safe birth at home - Concerns about ability of maternity service to be available for home birth when required - Perceptions that maternity services are under-funded/under-resourced to provide appropriate level of care
	Continuity in care	Continuity	- Concerns that would not receive adequate aftercare (including breastfeeding support)
Perceptions of birth as a ‘natural’ event	Perceptions of ‘Natural Birth’	Assistance	- Differing perceptions/meanings related to ‘natural’ birth linked to level of intervention
		Place	- Differing perceptions/meanings related to ‘natural’ birth linked to place (hospital vs home)
		Medical technology	- Differing perceptions on the role of medical technology in having a natural birth (safe/clean vs intrusive)
Sources of information for women	Sources of information for women	Mode	- Mode of information - Information from people valued more than information leaflets
		Credibility	- Role of informants matters (having professionals experience of home birth and ‘like minded’ people are seen as credible)
		Formality	- Influence of formal routes (hospital antenatal classes, hospital tours)
		Informal Prioritization	- Role of family, friends in providing information - Home birth not prioritized
The role of health care professionals	The role of health care professionals	Credibility	- Role of Health professionals – seen as credible source of information
		Gatekeepers	- HPs described as consciously and unconsciously controlling (restricting) information about place of birth
		Influence	- HPs seen as explicitly and implicitly influencing decision-making (skewed to OU as place of birth)
Women’s responses to the offer of choice	Perception of choice	Choice – realities	- Preferred choices not always available (e.g. alternative options not given, or unable to be delivered in practice)
		Choice – acceptance	- Extent to which lack of choice is perceived by women to be acceptable (e.g. most women accepting of limited choice)
		Choice- skills	- Exerting choice for home birth requiring special skill sets e.g. ‘confidence’ and ‘motivation’
	Response to choice	Responsibility	- Choice experienced as a responsibility (unwanted by many)
		Rights to choose	- Choice sometimes needs to ‘fought’ for if it does not align to ‘usual’ routes
		Support	- Lack of support experienced for choices made (from family, health professionals)

The initial coding framework was agreed by JNS, KS and SK, based on the research aims and previous literature. This was applied to data from each of the focus groups by JNS according to its meaning and content. The results of this deductive coding strategy were then discussed with KS and SK (who had familiarised themselves with the data). Data that did not fit within this framework was discussed and a final coding framework agreed that was then applied across the whole data set by JNS. Related codes were grouped together and labelled to form descriptive themes, with a summary written for each. The final ‘interpretative’ step of analysis involved all members of the team working together to understand how participants attached meaning to home-birth. This involved generating analytical themes which inferred implications for service provision from participants’ descriptions of their lived experience. Service users were not involved in the interpretation of the findings.

Several strategies were employed to maintain trustworthiness in the analysis, including keeping a clear and transparent audit trail, writing a reflexive diary, and discussing emerging understandings within the research team. We also reflected on how our positions as health service researchers with a background in maternity care and social sciences could influence the analysis and challenged preconceptions in our discussions in an effort to remain open to unexpected discoveries.

JNS was a clinical research student and midwife at the time of the research. All other authors are experienced qualitative health services researchers. SK, AH and BT are clinical academics (midwife, nurse and public health physician respectively).

## Results

### Participant characteristics

Data was gathered from 28 participants who attended 5 focus groups with between 2 and 8 members (2, 5, 6, 7 and 8 participants). Twenty one (75%) having a single child, and the remaining 7 (25%) having 2 children or more (see Table 2). The largest ethnic group was white, making up 16/28 of the participants, followed by Pakistani (*n* = 4), and Black or Black British (*n* = 3). Of the 26 women who disclosed their age, 15 were aged between 16 and 29 yrs., and 13 were over 30 yrs. old, with a mean age of 29 years and 9 months. At two groups fathers were present (but not as research participants), and one father translated for his wife who had limited English language skills.

**Table 2:** Participant characteristics

Variables		*n* = (%)
Ethnic Origin	White	16 (57.1)
	Mixed	1 (3.5)
	Black or Black British	3 (10.7)
	Asian or Asian British – Indian	1 (3.5)
	Asian or Asian British – Pakistani	4 (14.3)
	Asian or Asian British – Bangladeshi	1 (3.5)
	Asian or Asian British – Any other Asian	1 (3.5)
	Other (Egyptian)	1 (3.5)
Age (years)	16–29	14 (50)
	30+	12 (42.9)
	Did not disclose	2 (7.1)
	Mean age (disclosed)	29 yrs. 9mths
Parity	1	21 (75)
	2	5 (17.9)
	3+	2 (7.1)

### Themes emerging from the data

Several themes relating to women’s perceptions of home birth emerged from the data: a lack of knowledge about what home birth looked like, the expectation that birth would take place in an Obstetric Unit (OU), trust and confidence in the maternity system, and perceptions of birth as a ‘natural’ event, . Two themes emerged regarding the influences on women’s choices: clear information provision, and the role of health care professionals. A final theme concerned women’s responses to the offer of choice. Data extracts are presented alongside participant details. In some extracts it was not possible to identify which participant was speaking, due to background noise in a busy environment where children and babies were present.

### What does home birth look like?

For many participants, home birth was an unfamiliar concept. If they did consider it as an option it appeared they had little knowledge on which to base an informed choice:

*‘I don’t even know if you have a home birth where do you give birth? Do you choose a room; do you…in a bed? I’ve got absolutely no idea’* Participant 4, 32, LR’

A few women wanted a water-birth, but this was not perceived as practical or affordable at home:

*‘I always wanted a water-birth, so I knew a home birth wouldn’t be feasible. I know you can but it’s a lot of hassle’* Participant 8, 32 HR

There were some women who were concerned about the mess and responsibility for cleaning up following birth:

‘*I wouldn’t want to be sitting in a pile of guts on my living room carpet and then cleaning it up’* Participant 9, 35 HR

There was a difference of opinion between 2 women about the memories having a baby in their house would leave:

*‘My husband was born at home, it’s always been talked about in a positive way. I think there’s something quite nice about the fact he was born at home’* Participant 8, 32, HR

‘*I didn’t want a home birth because I didn’t want to walk into a room and think I had my baby there…the reality is it wasn’t a pleasant experience to remember and I’m really glad I didn’t have that experience at home’* Participant 10, 32, LR

Some of the women from minority ethnic backgrounds questioned the practicality of home birth because of the lack of availability or willingness of relatives to support the mothers during and immediately after birth:

‘*And what happens with the mother-in-law, does she have to get involved with the birth and clear up afterwards?’* Participant 5, 28, LR.

Immediate postnatal care was also something that concerned many of the participants, as they were unsure about what would be provided:

*‘Is there a dedicated length of time that people spend with you before they leave you for their next customer? The last thing you want is …you’ve got this baby and you’re at home sitting in the mess and they say ‘Right okay, well, I’ll see you in community in a weeks’ time’* Participant 9, 35, HR

Several women welcomed the prospect of one-to-one care from the Midwife at home births, although this was usually related to a poor experience in an OU:

*‘When you’re having a home birth you’ve got that attention haven’t you, they don’t have to rush out of the room to go to someone else’* Participant 11, 23, LR

Some of the women suggested that perhaps more could be done to provide information about the reality of home birth, including the use of television documentaries, and placing televisions in antenatal clinics which could feature information films. However overall there were mixed perceptions concerning the nature of home birth.

### Expectation of birth in an OU

Most women talked about their ‘assumption’ that they would give birth in an OU, often recalling that no other option was mentioned:

‘*It was assumed by everybody like myself and my loved ones that it would be in hospital… it wasn’t really discussed’* Participant 1,29,LR (Low-risk)

Strong cultural norms also played a part:

‘*I’ve never heard of it* (home birth) *it’s not common, none of my family or my sister have had that experience or anyone I know has needed to consider it’* Participant 2 24, LR

Many women felt that birth carries significant risk and is safest when it takes place in an OU, identifying their main concern being an ‘emergency’ or ‘something going wrong’. There was a strong desire to have specialist medical support nearby, regardless of risk status.

*‘if all goes wrong I’m a trolley away from a C-Section if necessary’* Participant 3,40,HR

Women reported that they only considered the hospital for their first baby, because of the associated risk:

‘*As a first time mum I would never have had a home birth because I didn’t know what was coming, I wouldn’t have felt safe enough*’

Participant unidentified, Focus Group 1.

While most participants were aware that they had a choice, they interpreted this as deciding ‘which hospital’ to give birth in and considered locality, familiarity, reputation and where their friends/family had received care, when making their choice:

*‘My husband did some research on google and found Trust A had a good reputation’* Participant 3, 40, HR.

Choice appeared to be influenced by ethnicity, with women who classed themselves as white being more aware of the choices available to them than women from ethnic minorities (42.9% of participants) who reported they were not offered a choice of location for birth, for example the following quote was from a Bangladeshi participant.

*‘I didn’t think you were allowed that, they didn’t mention that’* Participant 5, 28, LR

Women from ethnic minorities were most likely to shift their perception of the practicality and desirability of homebirth by the end of the session, illustrated in the quote below from an Egyptian participant, while this was not observed in many women from a white background:

*‘Only in critical cases should go to hospital. It is the nature of people. Nature is best’* Participant 6, 25, HR (Egyptian).

### Trust and confidence in the maternity system

Several participants indicated they had doubts that maternity services could support a safe and responsive home birth. In many cases this related to a perception that the resources necessary to provide such a service were lacking.

*‘I just don’t think there’s enough money. I don’t think it would happen. It’s just so underfunded and not enough staff. I just couldn’t trust it I wouldn’t’* Participant 4, 32, LR

A few women, some with experience of home birth, and others who were contemplating it, reported how women were asked to come into hospital during labour because no midwives were available, which made them reluctant to choose home birth again:

*‘Obviously we’re often told “Oh yeah, it’ll be available.” And often they say to you, “You’ve got to come into the hospital I’m afraid”’* Participant 14, 28, HR

Some women also reported hearing stories about babies being born before the Midwife arrived:

*‘That’s the only anecdotal stories I’ve heard about home birth, the gas and air hasn’t turned up, the Midwife hasn’t turned up….’* Participant 15, 39, HR

The majority of women reported that the aftercare provided in the hospital was good, and suggested that this would not be replicated at home. This included breastfeeding support, and a constant professional presence for reassurance and help if needed:

*‘But then what do I do with his first nappy, because it was just like tar. They were brilliant. So if you have that level of support in the home who’s available, who’s on the end of the phone, but more importantly who’s staying there while you get those questions done?* Participant 3, 40, HR

### Perceptions of ‘natural birth’

The majority of women who participated in this study considered birth to be a ‘risky’ event. Women who had given birth vaginally suggested that that they would have confidence in their ability to achieve a ‘natural’ birth in the future.

The women shared different definitions of ‘natural’ depending on their preference of birth setting. The overwhelming majority felt that the availability of medical technology secured a ‘safe and clean’ birth.

‘*Hospital is the natural thing’* Participant 5, 28, LR.

The fact that this participant saw the hospital as the ‘natural’ thing, suggests that the notion of hospital birth is inculcated so completely that she was unaware of the inherent contradiction of this comment, as hospitals are places where technology and medicine prevail. Women, who believed in their ability to give birth outside of the OU, used the term ‘natural’ in a way that reflected an awareness of this:

*‘More natural not having machines…constantly beeping’* Participant 6, 25, LR.

### Sources of information for women

The participants reported a range of ways they had obtained information about home birth. It was generally by informal means, and through involvement in antenatal classes and meeting with groups of ‘like-minded people’.

Many women stated that, after friends and family, antenatal classes were the main source of information, although the ‘hospital tour’ was highlighted as a rich source of knowledge to support decisions:

‘*They took us round delivery suite and the birth centre, but I don’t remember anything about home birth’* Participant 11, 23, LR

The importance of having ‘like-minded’ sources of support, with whom women can identify is illustrated in this extract:

*‘I went to some of those pregnancy yoga classes – ‘hippy dippy ones’ and probably it wasn’t for me, but I know a lot of them were either considering or having home births. And I just didn’t feel it was for me*. Participant 12, 33, LR

Some participants’ described how place of birth was simply one of many pieces of information shared, and that it was not detailed or prioritised:

*‘My Midwife did have a vague discussion about place of birth towards the end of my pregnancy’* Participant 12, 33, LR.

It was reported that leaflets were not well-regarded or used, except for two participants who did not have friends or family to advise them:

*‘I was given a leaflet by somebody, but I confess I didn’t really read it’* Participant 12, 36, LR

The need for information to be conveyed by people with the necessary experience was also identified:

*‘I think it would be nice to meet someone who did home birth, not just a person who gave birth but someone who also was there and delivered because you get a little information’* Participant 16, HR.

### Role of health care professionals (HCPs) in influencing decisions on choice of place of birth

The data suggest that professionals, including midwives, have little influence on women’s choices, with friends, family, antenatal classes (usually delivered by trained childbirth educators) and the media identified as being the main sources of information:

*‘I think I was aware of home birth as an option, but certainly not from a health care professional’* Participant 12, 36, LR.

There was, however some evidence of hostility from HCPs if their expectation of control was challenged:

‘*They didn’t really want me to have one* (home birth) …*There was quite a lot of negativity. And in fact even though my husband phoned to say I was in labour they said ‘we haven’t got the staff. Can you get into hospital’….I just felt no-one approved’* Participant 14, 28, HR

There were many reported examples of midwives restricting women’s choice by not presenting all the alternatives:

*‘They just gave us directions how to get there, we weren’t given any options’* Participant 17, 29, HR

There was one example of a midwife presenting home birth differently to other options suggesting non-verbally that it was not an option she would recommend:

‘*We knew each other anyway as I had the same Midwife she said I could have delivery suite or birth centre, or you can have home birth…then she looked at me and smiled* [participant impersonates midwife with a wry, derisory expression] *and said ‘Yes well…..’* Participant 9, 35, HR.

Two women reported seeking advice and guidance from a midwife but in the end taking the decision not to pursue a home birth.

*‘They did give me the option of a home birth because we were fine all the way through. I remember talking to her (midwife) about it and she seemed to want me to do it but I didn’t fancy it at the time.’* Participant unidentified, Focus Group 2

### Response to being provided with choice

A few women reported changing their choice of birth place in order to access particular birth options, however this active choice was only reported by white British participants:

*‘Trust A doesn’t allow you to labour in water* (after previous caesarean section) *so I switched to Trust B’* Participant 15, 39, HR

Other participants reported how their preferred options were not available, and while they expressed some frustration, they described how they accepted what was on offer:

‘*I chose hospital birth and to have a waterbirth but couldn’t the first time as my blood pressure was too high and the second time it (the birthing pool) was being used’*, Participant 16, HR

One woman acknowledged that although she had been offered a choice and exercised it, she felt the weight of responsibility this placed on her:

*‘It felt quite a strange process to be the person controlling it…it’s quite complicated actually, being given the choice was almost something you didn’t want as a pregnant woman. Because the last thing you want is to make a choice to the detriment of your child’* Participant 15, 39, HR

## Discussion

Many of the themes identified in this study confirm findings from earlier work. For example, most women in this study assumed they would give birth in an OU, which has been observed elsewhere. [16, 21–24] The perception of birth as a high risk process and the influence of cultural norms on choice have also been identified in earlier work [16, 21, 22, 25, 26]. Similarly the different views of ‘natural’ birth and control have been identified previously [16, 21, 25, 27–29]. The white women in our study seemed to be aware that they had a choice, and reported they were more likely to actively choose their place of birth, which is consistent with earlier studies. [21, 25, 28, 29] However an important new finding from our study was that women from minority ethnic backgrounds expressed an interest in home birth when they realised it was an option.

Some women in our study actively sought to access particular options, while others were frustrated at having to accept the options offered. This is consistent with the ‘active chooser’ and ‘adaptor’ categories identified by Pitchforth et al. [23]. It was also reported that making a choice was an onerous process because of the potential implications of getting it wrong, also noted elsewhere. [23, 30] Our finding that white British women were more aware of their choices is consistent with previous work which found that women from minority ethnic groups are less aware that were choices, highlighting a deficit in the information about home birth provided for these women. [21, 25, 28, 29] There were many examples suggesting that women valued the information sharing and group support they experienced in antenatal classes and meeting with groups of ‘like-minded people’, which has been noted by Catling-Paul et al. [31]

Our study is consistent with others’ work which found some women construct a narrative around place of birth choice, synthesising advice and guidance from professionals with their own knowledge. [21, 29] The literature and this study also describe the negative influence that professionals can have on facilitating genuine choice for women when considering place of birth. [16, 25, 32, 33] Professionals’ attitudes have been observed to influence women’s choices and behaviours elsewhere in maternity care. Dominant cultural norms can restrict the choices offered to women in prenatal testing. Professional practice norms and the adoption of ‘Baby Friendly’ practices have been shown to increase the duration of breastfeeding [34]. Choice of maternity provider by women (which health centre, rather than hospital or home) increases when respectful provider attitudes are demonstrated [35].

Midwives have been found to be much more supportive of home birth than medical staff, [36, 37] and in the UK women who are most suitable for home birth are generally cared for by midwives. However, even among midwives this support is often conditional, with barriers including confidence, knowledge, training, staffing, cultural norms, and whether they are required to attend home births [36, 38, 39]. Exposure to home birth in education and practice has been associated with favourable attitudes to home birth among health professionals [37]. In addition, training and support have been shown to improve the knowledge and confidence of midwives in discussing place of birth options with women [38]. Maternity services should consider providing more experience of home birth for midwives and student midwives, and specific training and support interventions. This would increase professional knowledge with a view to eliminating this barrier to choice. This would be best undertaken in an organisational and professional culture which supports choice, for example by providing adequate staffing of services, and championing of home birth among obstetric and midwifery staff.

There was little confidence in the ability of maternity services to provide home birth among a number of participants in our study. As discussed earlier in relation to professional influence, previous work has also identified staffing pressures as a barrier to midwives promoting home birth to women, due to concerns about ability to provide equitable maternity care in a pressurised system, [39] and women have expressed fears that midwives will not be available to attend in labour [40].

The data in the ‘*What does a home birth look like?’* And the *‘Trust and confidence in the maternity system’* themes reveal some new insights on women’s perceptions of home birth. An important new finding from our study was that women reported that they did not know what home birth looked like. They often had little knowledge, and were concerned about mess, cleaning, the responsibility of relatives, and what level and type of support would be provided, and for how long. Women had particular concerns about the availability and quality of postnatal care. Some women did not like the idea of giving birth to a baby in their home, while others welcomed the one-to-one care a home birth offers. A lack of exposure and information, plus misinformation and uncertainty about home birth was evident, and the findings suggest that whatever services are doing at present to inform women about their birth place options is not meeting all of women’s information needs. This reflects the complex challenges involved in determining the type of service provision required by women, even in a specific, albeit diverse, area.

In all of the focus groups, when home birth was first discussed, predominantly negative views were expressed. However women from ethnic minorities, whether in homogenous or heterogeneous focus groups, reported an increased awareness of the practicality and desirability of home birth by the end of the session, which was not the case with many women from a white background. This suggests that the opportunity to discuss home birth may specifically encourage more women in minority ethnic groups to consider it as a realistic option for them, and that a disparity in knowledge and access to information about home birth may be a particularly important restriction of choice for women from minority ethnic backgrounds. There are parallels here with evidence from the United States, where women of colour are much less likely to choose home birth [10], more likely to be dissatisfied with their involvement in decision-making [41], and African American women are likely to express interest in home birth for future pregnancies [42]. Midwives and other maternity care providers should be supported in providing all women with meaningful information to aid birth place decision-making. Midwives in the UK have been found to shape their discussion of place of birth choices based on their perception of different groups of women’s openness to the various options [38]. This could be addressed by working with midwives to address their cultural competence, and challenge their assumptions about women in different groups, which also apply to other aspects of care beyond birth place decision support [43].

Coxon et al. developed a conceptual model of the experiences of choosing, preferring or deciding where to give birth from their evidence synthesis, which describes influences on birth place choice arising from women, and from services [16]. While our empirical work has identified some new influences on birth place choice, these new findings are consistent with a number of domains in the conceptual model [16]: women’s pre-pregnancy beliefs about birth (e.g. they think that home birth will involve cleaning up mess); women’s previous experiences of birth and birth settings (e.g. they have no experience of home birth so there is uncertainty); staff (lack of) provision of information to women about choice of place of birth (e.g. women do not know what happens at a home birth, therefore it may be the case that midwives do not provide this information).

A key challenge in informing women about home birth and increasing choice is the lack of exposure to home birth in day-to-day life, as it is so rare [9]. Compared with hospital settings, which are the norm, women have fewer opportunities to acquire knowledge about home birth from family, friends and the media, and so women do not know ‘what home birth looks like’. This could be mitigated by providing information during maternity care, for example in routine antenatal appointments, by a midwife. Additional activities might include opportunities for pregnant women and their families to meet other women who have had home births, and/or midwives who provide home birth care, to share information and experiences, and promote discussion. Print and digital resources, along with appropriate signposting, are further sources of information, for example the Birth Choice tool provided by Which? in the UK [44]. These measures may be particularly important for women from ethnic minority groups, as they appear to have greater information needs. Women are likely to require some degree of tailored individual support to make an informed choice. A further means of addressing this could be to provide information via the broadcast media, such as drama and reality television outputs. In a 2016 scoping review of the literature regarding the media portrayal of birth, Luce et al. found that it appears to influence how women think about birth [45]. They also found that birth in the media was often dramatic and medicalised, with fewer representations of normal birth, and highlighted a need for more portrayals of low risk, ‘uneventful’ birth. In addition to health professionals changing the way they present birth options, changes to media portrayals could be key in changing perceptions and filling the gaps in women’s knowledge. Indeed a number of the respondents suggested the wider use of television documentaries and the provision of information films about home birth in ante-natal clinic waiting rooms would be helpful.

When planning the study the intention was to explore the accounts of women designated as low-and high-risk women’s and compare them. However during the conduct of the focus groups, the researchers were not definitively aware of the risk status of the women, as a demographics form was completed anonymously at the end of the session. Although the risk status of some women became obvious during the group as the women referred to their birth experiences, these did not appear to define the women’s views or their ability to consider all places of birth, whether or not they were appropriate to them. It became clear that the risk status of the women was largely irrelevant to the majority of the participants’ views, as women who were low-risk up until they gave birth, and then, for instance, had a caesarean section, could clearly and articulately recall their decision making processes during their pregnancy concerning place of birth choice. The themes encompass the accounts of women deemed to be high and low risk.

### Limitations

Individual interviews with women may have provided richer data, though the use of focus groups was particularly beneficial in revealing that women’s perspectives are shaped and changed by meeting and discussing options in a group setting. Although the characteristics of the sample may limit the transferability of the findings to other populations, the diverse membership suggests the findings are likely to reflect key concerns of women in this situation. As all the women in the study had given birth before, it was difficult to determine whether they, or nulliparous women were most open to the idea of alternative places of birth as identified by Coxon et al. [21] The study setting means that some women’s perspectives, such as those who do not attend community groups, and those in non-urban settings, were not gathered.

The presence of babies and children in the groups interrupted the discussions at times, but we feel conducting the groups in this natural, comfortable environment with no need for childcare increased participation and generated rich insights in their experience. Some of the women in the one focus group where men were present as interpreters contributed less to the discussion once men joined the group. It is possible that this limited the data collected. However, it also highlighted important cultural issues that need to be factored into approaches to support women with home birth. Two of the researchers are midwives, which may have affected the approach to gathering and interpreting the data, though the team actively reflected upon the impact of professional experience and perspectives on the data collection and analysis. All members of the research team are white British, and the team acknowledged and reflected on this in the interpretation of specific findings relating to ethnicity and minority groups.

Despite these limitations this study provided new detailed information about women’s perspectives on home birth. Accounts from a wide range of women in a large multi-ethnic city were collected and the qualitative approach provided rich, in-depth data. It was informed by prior literature review to locate findings within, and build upon existing knowledge in this area. It appeared that the findings approached saturation (i.e. no new themes) [46] suggesting that they can be extrapolated beyond the participants in the focus groups.

## Conclusion

Whilst consistent with earlier work [16, 21–33, 39, 40], the study reported here uncovered some important new findings. First, women have a lack of understanding of what home birth entails, suggesting that new approaches are needed in order to inform choice. Second, our study indicates that when given the opportunity to discuss it, women from minority communities may consider home birth. This suggests services should consider providing and facilitating opportunities for such discussions. It appears that many women may be missing out on the opportunity to consider home birth. The findings from this research can be used to develop approaches that meet women’s needs and inform genuine choice.

## Additional file

Additional file 1: Focus group topic guide. (DOCX 392 kb)

## Abbreviations

HCP: Health care professional
HR: High risk
LR: Low risk
NHS: National Health Service
OU: Obstetric Unit
