# Supplementary material for: ‘I didn’t think you were allowed that, they didn’t mention that.’ A qualitative study exploring women’s perceptions of home birth
Source: BMC Pregnancy Childbirth. 2018 Apr 18;18:105. doi: 10.1186/s12884-018-1733-1 (PMC5907292; doi:10.1186/s12884-018-1733-1)
Supplement: Supplementary file 1 — Focus group topic guide. (DOCX 392 kb) [file 12884_2018_1733_MOESM1_ESM.docx]

## FOCUS GROUP TOPIC GUIDE - WOMEN

| **Facilitator’s welcome, introduction and instructions to participants:**  Welcome and thank you for agreeing to take part in this focus group. You have been asked to participate as your point of view is important. |
| --- |
| **Introduction:**  This focus group discussion is designed to understand your views on home birth and to explore if there is any more information or support you would like, to think about having your baby at home. The focus group discussion will take about an hour, however if you need to attend to your baby, use the toilet or leave early please feel free to do so. We are not expecting a fire alarm practice today, however if the fire alarm sounds please leave by the doors marked fire exit.  We will record the discussion and once transcribed the all the participant’s names will be removed so quotes from the discussions can be used in reports but no-one involved will know who said what. If you change your mind about taking part in the study you can do so without giving a reason, however due to the nature of the focus group your data will still be used up to the point of withdrawal but no direct quotations will be used. |
| **Confidentiality:**  We and the other focus group participants would appreciate it if you would refrain from discussing the comments of other group members outside the focus group. If there are any questions or discussions that you do not wish to answer or participate in, you do not have to do so; however please try to answer and be as involved as possible.  How the group will run:   - So we don’t miss any important points, we ask participants to speak one at a time. - There may be a temptation to jump in when someone is talking but please wait until they have finished - We are interested in your views, so there is no right or wrong answer - You do not have to speak in any particular order - You do not have to agree with the views of other people in the group, in fact we are very interested in the range of different views on home birth. - Does anyone have any questions? - Ok let’s begin |
| **Warm up – ice breaker**   - It would be very helpful if everyone tells us their first name and if your baby has done anything new or funny in the last few days |

| **Introductory question**  I am just going to give you a couple of minutes to think about your views on home birth. Is anyone happy to share what their immediate thoughts are? |
| --- |
| **Guiding Questions**   1. Did you think about the different places you could have your baby? 2. Did any health professionals discuss choice of place of birth with you?   - If so who and at what point during your pregnancy?  - Was it helpful? Did you need any more/different information?   1. Did you discuss where you intended to give birth to your baby with family and friends?   - Did their views influence your choice?   1. What factors did you consider when you chose to have your baby/babies either in hospital or at home? (e.g safety of both you and your baby, surroundings, knowing who was going to look after you) 2. Do you think there are any good things about home birth? If so what to think they are? 3. Is there anything that you feel is worrying or negative about home birth? 4. Can you think of any information you would need to make you consider home birth in the future?   -How would this information best be communicated to you? (e.g. discussion/leaflets/dvd’s/socialmedia/one-to-one or group discussion)   1. What would be important to you that a Home Birth Team offers? (e.g. /known midwife/1-1 support in labour/guarantee to attend/removal of clinical waste) |
| **Concluding question**  Of all the things we’ve discussed today, is there one thing about your experience of pregnancy and child birth that would make you consider a home birth if you had future children? |
| **Conclusion**   - Thank you for participating. - Your opinions will be very useful in informing the home birth team the kind of service you want. - We hope you have found the discussion interesting - If there is anything you are unhappy with or wish to complain about, please feel free to speak to me at the end of the group or contact me via telephone or email - We would like to remind you that any comments featuring in this report will be anonymous, and it will be appreciated if any opinions expressed are not discussed outside of this group. - We will be sending you a summary of the views expressed today and at other focus groups, your thoughts and comments will be very welcome |

Thank you
